# An exploratory pre–post study of an intensive somatosensory activity-based intervention on participation-related goals, motor performance and somatosensory function in children with unilateral cerebral palsy

**DOI:** 10.3389/fped.2026.1862592

**Published:** 2026-06-25

**Authors:** Patricia Jovellar-Isiegas, Luis Enrique Roche-Seruendo, Diego Jaén-Carrillo, Manuel Gómez-Barrera, César Cuesta-García

**Affiliations:** 1 Faculty of Health Sciences, University of San Jorge, Zaragoza, Spain; 2 Department of Sport Science, University of Innsbruck, Innsbruck, Austria; 3 Department of Occupational Therapy, Centre for Advanced University Studies La Salle, Autonomous University of Madrid, Madrid, Spain

**Keywords:** unilateral cerebral palsy, occupational performance, motor performance, participation-related goals, somatosensation, somatosensory activity-based intervention, occupational therapy

## Abstract

Somatosensory impairments are common in children with unilateral cerebral palsy and may be associated with limitations in upper-limb function and daily activities. The aim of this exploratory single-arm pre–post study was to examine pre–post change following a novel intensive somatosensory activity-based intervention (ISABI) targeting the more-affected upper limb, focusing on goal-related occupational performance (primary outcome), upper-limb motor performance, and somatosensory function. Sixteen children aged 6–15 years completed 36 h of intervention over three weeks (30 h clinic-based and 6 h home practice). Outcomes were assessed pre- and post-intervention using the Canadian Occupational Performance Measure (COPM; performance and satisfaction), the Box and Block Test, the Jebsen–Taylor Hand Function Test, and a predefined somatosensory assessment battery covering tactile registration, tactile spatial perception (unilateral and bilateral), graphaesthesia, haptic perception (stereognosis of objects and forms), texture perception, and functional sensibility. Occupational problems were most frequently related to self-care (81.25%), followed by leisure (12.50%) and productivity (6.25%). COPM performance increased by 71.47% and family satisfaction increased by 62.51%. Manual dexterity and hand function improved by 12% (Box and Block Test) and 12.38% (Jebsen–Taylor Hand Function Test), respectively. Somatosensory outcomes showed improvements in several domains, including unilateral and bilateral spatial discrimination, graphaesthesia (33% increase), texture perception (33% increase), and functional sensibility (18.52% increase), while tactile registration and stereognosis showed limited change. These exploratory findings suggest potentially meaningful pre–post changes in goal-related occupational performance and satisfaction, upper-limb motor performance, and selected somatosensory outcomes following ISABI in children with unilateral cerebral palsy. Given the exploratory single-arm pre–post design (no control group and no follow-up), findings should be interpreted cautiously and require confirmation in adequately powered randomised trials with blinded assessment and longer-term follow-up. NCT04235088.

## 1 Introduction

In children with unilateral cerebral palsy (UCP) the performance of the more-affected upper limb (UL) is considered as the main limiting factor hindering children's successful participation in activities of daily living (ADLs) (1). Moreover, recent studies show that more than 75% of children with UCP have impaired somatosensory processing in the UL (2–4), which significantly impacts motor performance (5, 6). Importantly, somatosensory impairments may not be confined to the more-affected UL, and reduced somatosensory function has also been reported in the less-affected (“unaffected”) limb in children with UCP, which may further influence functional use and participation (3, 7).

Somatosensory function can be clinically organised into three related levels: tactile processing, proprioceptive sensitivity, and higher-order cortical/haptic functions (8). Tactile processing refers to the registration and perception of cutaneous stimuli and includes modalities such as light touch, pressure, vibrotactile input, temperature, pinprick, tactile localisation, two-point discrimination (2PD), and bilateral simultaneous stimulation, depending on the clinical assessment framework used (8, 9). Proprioception comprises information about body and limb position, movement, and force-related aspects, arising from muscles, tendons, joints, ligaments, and skin (8, 10). Higher-order cortical/haptic functions, including stereognosis or haptic object recognition, require the integration and interpretation of primary tactile and proprioceptive afferent input (8, 11). This distinction is particularly relevant in children with cerebral palsy, in whom impairments may affect tactile registration, tactile perception, proprioception, and haptic object recognition, with potential consequences for manual exploration, UL function, and participation (12).

Somatosensation is essential for the development of the fine motor control required for functional hand use (13). It enables effective exploration of objects and interaction with the environment. Beyond basic registration and discrimination, these inputs help build a coherent internal representation of the body and objects, support the planning of purposeful actions, and enable adaptive interactions with the environment during everyday tasks (14, 15). Therefore, somatosensation has the potential to support participation in daily and meaningful activities, as well as overall occupational performance (2, 16).

The feedback and guidance needed to correct errors in motor actions depend on the quality of these sensory inputs, which is essential for successful motor task performance (17). From a motor control and motor learning perspective, somatosensory feedback contributes to calibrating force, timing and movement trajectories, detecting mismatches between expected and actual sensory consequences during practice, and updating internal representations that support skill acquisition and retention (18, 19). Importantly, the role of somatosensory processing in supporting motor performance is not unique to cerebral palsy but reflects general principles of sensorimotor control observed across populations.

It is known that there are disturbances in the way children with cerebral palsy process sensations (20, 21). It has even been suggested that deficits in somatosensory processing may result in learned non-use on the more-affected side (13), assuming the implications that this might have for adequate performance in bimanual tasks. It has also been observed that difficulties in proprioceptive processing contribute to poorer performance of children with UCP in ADLs (21). In summary, the contribution of somatosensation to motor function suggests that interventions that target somatosensation may have the potential to improve motor performance (22).

Intensive activity-based and goal-directed therapies currently show the highest level of evidence in improving UL motor function in children with UCP (23). These therapies are based on the principles of motor learning and share some key components such as repetitive and intensive practice, gradual increase of task challenge, and feedback on performance (24). This is consistent with recent clinical frameworks emphasising motor learning principles underpinning bimanual therapy and constraint-induced movement therapy in UCP (25). Goal setting in collaboration with the family is another essential ingredient for success (26). These motor-learning-based therapies have demonstrated neuroplastic changes associated with improved motor function (27). In some studies, tactile performance has been assessed as a potential by-product of motor training (28–30). However, none of them demonstrated significant improvements in tactile performance. This limited transfer is expected: even with high doses, tactile stimuli experienced “unintentionally” during motor practice are likely to yield limited or no tactile gains (31).

Tactile learning depends on modality-specific conditions that are not systematically addressed in motor-focused, goal-directed training, including consistent stimulus contact/placement and appropriate timing, as well as attention explicitly directed to sensory features while the motor action is being executed (31). Without structured sensory experiences and instructions that prioritise tactile discrimination, the child's attentional resources are often allocated primarily to achieving the motor goal, reducing opportunities for perceptual learning (31). Findings from subsequent studies comparing tactile function before and after motor-oriented interventions are consistent with this interpretation (32, 33).

This gap provides a rationale for targeted somatosensory interventions. The key learning-dependent principles underpinning effective motor training are also those driving neural plasticity (22, 34). It seems reasonable to argue that learning-dependent neuroplastic changes in somatosensory brain regions may occur when these same principles are applied to interventions focused on somatosensory activities. Furthermore, somatosensory pathways, although disorganised, remain active and are likely responsive to treatment (35).

Regarding evidence-based interventions addressed to improve somatosensory function, the latest systematic review (36) found none for children with UCP and, consequently, the need to develop those interventions was indicated. From that review to the present day, some studies have shown promising but not yet conclusive results (37, 38).

Somatosensory discrimination, particularly tactile and proprioceptive discrimination abilities, appears to be modifiable through intervention and may contribute to improvements in bimanual performance in children with UCP (37). For this reason, therapists have been encouraged to systematically assess somatosensory function and to incorporate sensory-focused intervention strategies when clinically relevant (39, 40). However, although the modifiability and potential functional relevance of somatosensory discrimination are increasingly recognised, it remains unclear how specific somatosensory impairments manifest in children with UCP and how these difficulties are associated with functional outcomes. Consequently, exploratory studies are warranted to better characterise these clinical patterns and to generate preliminary evidence that can inform future research questions and appropriately powered study designs.

Therefore, the aims of this exploratory pre–post study of a novel intensive somatosensory activity-based intervention (ISABI) were:

(a) to assess pre–post change in goal-related occupational performance using the Canadian Occupational Performance Measure (COPM; performance and satisfaction) as the primary outcome; and
(b) to quantify pre–post change in secondary outcomes, including UL motor performance [Box and Block Test (BBT); Jebsen–Taylor Hand Function Test (JTHFT)] and somatosensory function of the more-affected UL, assessed using a predefined somatosensory battery (tactile registration, tactile spatial perception, bilateral tactile perception, graphaesthesia, haptic perception, texture perception, and functional sensibility).

## 2 Materials and methods

### 2.1 Study design and participants

An exploratory study was conducted with a single-blind design for assessors. The study comprised a single arm (UCP intervention group) with two assessment time points (pre- and post-intervention), and a 3-week intervention period. The protocol was registered at ClinicalTrials.gov (NCT04235088), and the study was approved by the Clinical Research Ethics Committee of Aragón, Spain (reference: PI19/230). The registry record was updated during manuscript preparation to reflect the final study information and protocol-related changes. The planned follow-up assessment could not be completed due to illness of the principal investigator; therefore, only pre–post outcomes are reported.

Participants were recruited from early care centres, associations, and rehabilitation centres in Aragón between September and December 2020. Families who volunteered to participate and met the inclusion criteria provided written informed consent prior to participation.

The inclusion criteria were: UCP diagnosis (congenital or acquired) confirmed by medical report; aged between 6 and 15 years; Manual Ability Classification System (MACS) (41) level I-III; Gross Motor Function Classification System (GMFCS) (42) level I–III, no botulinum toxin infiltration 4 months before the assessment pre-intervention or during intervention; no moderate nor severe cognitive impairment compatible with attending a special education school. Additionally, children who presented fractures and/or trauma in the UL in the last 12 months and children who had an orthopedic intervention in the 6 months preceding the study or during the study were excluded.

### 2.2 Procedures

#### 2.2.1 General intervention procedures

It was held at the Asociación para la Investigación en la Discapacidad Motriz (Association for Research in Motor Disability) and lasted for 3 consecutive weeks, every day from Monday to Friday for 2 consecutive hours per day (30 h of practice). During the weekends, somatosensory activities were explained and handed to each family with the proposal of playing together at home for one hour per day (6 h of practice). The somatosensory intervention was carried out in a group setting and the children were divided into two groups according to age (6–10 and 11–15 years old). Each child had a trained interventionist that was in charge of providing the treatment throughout the intervention. The team of interventionists were trained prior to the start of the study in order to have the necessary knowledge on how to administer the therapy accurately and to feel competent to carry it out. The lead researcher led the sessions and supervised the interventions to assure their uniformity, adjusting the somatosensory training protocol to the individual needs. In each session, different domains of somatosensory function were worked on. For more information on the details of somatosensory intervention and session design, see Supplementary Material 1.

A daily checklist, designed for that purpose, was used by the interventionists to register the gradually increased complexity of the activities and individual progression. In this checklist, all the domains of somatosensory processing, as well as the different variables for grading the complexity of the activities within each domain, were included. For more detailed information on the type of activities, the variables for grading the complexity of the activities and examples of materials, see Supplementary Material 1.

#### 2.2.2 Theoretical principles underlying the ISABI model

**Figure 1:**
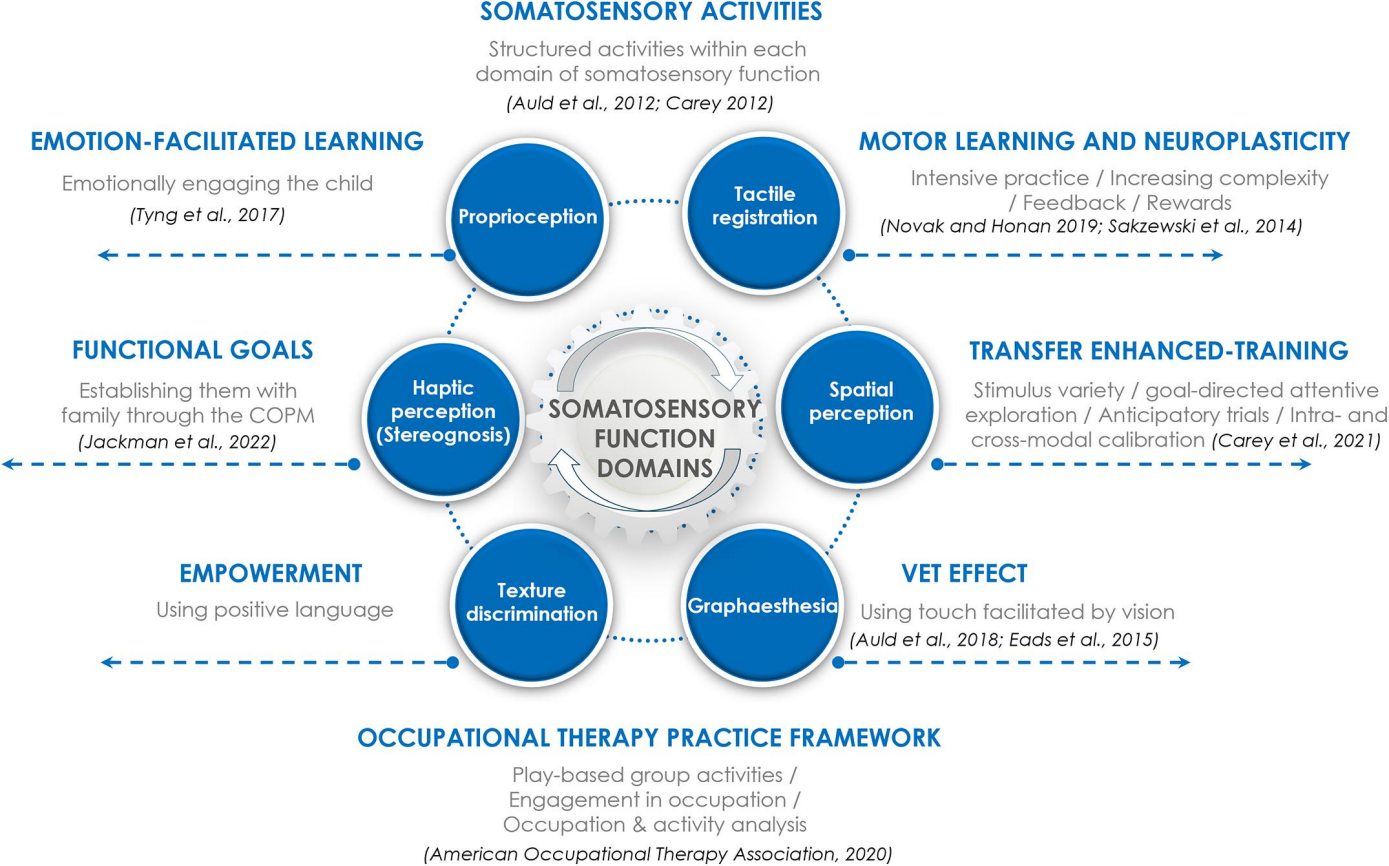
Theoretical principles underlying the ISABI model.

*Specially designed somatosensory activities approach*, for which an understanding of the complex nature of somatosensory function was essential. To develop the model we took as a reference the framework of tactile function proposed by Auld et al. (43). In order to have a more comprehensive view of somatosensation, the definition proposed by Carey et al. (44) was also taken into account (Figure 1).

*Principles of motor learning and neuroplasticity* (24, 45), such as intensity of practice, feedback, reward and structured practice with increasing complexity. To grade the progressive difficulty of the somatosensory activities, specific variables were used for each somatosensory function domain. See “Variables for assessing the complexity of activities” in Supplementary Material 1 for further details.

*Principles based on transfer-enhanced training*, known as sense© training (34). These were: variety of tactile stimuli, goal-directed attentive exploration of sensation without vision, deliberate anticipation trials, intra and cross-modal calibration of somatosensory perception as well as feedback on performance.

*Use of visually enhanced touch or the VET effect* (46) as proposed by Auld et al. in the Apartment Block Theory (31). This means asking the children to visually attend to their hands during tactile stimulation. In the ISABI model, the VET effect was used in the initial phases of each new somatosensory activity.

*Principles of the Occupational Therapy Practice Framework: Domain and Process* (47), recognising play as a core childhood occupation and linking engagement in occupation to choice, motivation and meaning within a supportive context. Accordingly, the ISABI model used themed weeks and play-based group activities to support motivation and sustained engagement during practice, with task demands graded using activity analysis to maintain an appropriate level of challenge while supporting successful performance within the group context.

*Empowerment through the use of language:* certain expressions used by children or families to refer to their more-affected side, such as “the sleeping hand” or “the bad hand” were replaced by “the powerful hand”. The language used by the therapists to refer to the more-affected side was always in positive terms.

*Setting functional goals agreed with the family* (26): on the first day of the assessment, two functional goals were set by each family, which were identified using the COPM.

*Emotion-facilitated learning:* Emotion has a substantial influence on cognitive processes, including learning, especially important in modulating selective attention, as well as motivation and behaviour (48). For this purpose, setting and creating an appropriate empathetic background using creativity and imagination, as well as the use of different communication aspects such as voice, look, gesture or dramaturgy were strategies specially taken into account by the interventionists during the sessions.

A detailed operationalisation of these theoretical principles—including brief definitions and concrete activity examples from the ISABI programme—is provided in Supplementary Material 2.

### 2.3 Assessment procedure

The children were assessed at the intervention site and the assessments were performed on two non-consecutive days. On the first day, the COPM (49) and Takata's play story (50) was conducted with the family and the motor tests were performed. During the second day, the somatosensory assessment battery was completed. For the motor and somatosensory assessments, the less-affected hand was tested first, followed by the more-affected hand. Exceptions were the Stereognosis of Familiar Objects (SFO) and Texture Perception tests, which were administered only to the more-affected hand. The assessments were conducted by two blinded pediatric rehabilitation clinicians (an occupational therapist and a physiotherapist), each with >5 years of experience in pediatric rehabilitation. Prior to data collection, both assessors were trained in all assessment procedures and completed a calibration session to ensure standardized administration and scoring.

#### 2.3.1 Primary outcome measure

The COPM was used as the primary outcome to assess goal-related occupational performance and satisfaction in daily life. The COPM is an individualised, client-centred outcome measure designed to identify and detect change over time in self-perceived occupational performance problems. In this study, two functional goals were identified collaboratively with each family at baseline. For each goal, child performance and family-reported satisfaction were rated on a 10-point scale, where higher scores indicate better perceived performance and greater satisfaction. The COPM is clinically relevant because it captures change in meaningful, participation-related goals and supports the evaluation of intervention outcomes in everyday contexts. Previous studies have demonstrated its validity, reliability, responsiveness, and usefulness in pediatric rehabilitation and in parents of children with disabilities (51, 52).

#### 2.3.2 Secondary outcome measures

UL motor performance was assessed using the BBT (53) and the JTHFT (54), the latter following the modified protocol reported by Charles et al. (55). The BBT assesses unilateral gross manual dexterity, whereas the JTHFT captures a broader range of timed unimanual hand functions relevant to activities of daily living. Both measures have shown good reliability for children with cerebral palsy (56).

A comprehensive assessment battery targeting multiple domains was completed taking into account current evidence-based recommendations related to somatosensory assessment (3, 12, 43). The battery included a tactile registration test (Semmes-Weinstein Monofilament), two unilateral spatial perception tests [Single Point Localization (SPL) and 2PD], a bilateral spatial perception test [Double Simultaneous (DS)], a spatial-temporal characteristics test (Graphaesthesia), haptic perception (SFO and Manual Form Perception Test), a Texture Perception test and a test to measure the concept of functional sensibility. The exact procedures implemented for each test followed the previously published protocol (3). To keep the main Methods section concise while ensuring reproducibility, administration, scoring, laterality assessed, impairment classification, and key clinimetric/psychometric notes for all motor and somatosensory outcome measures are summarised in Supplementary Material 3. Impairment classification was applied only to Semmes–Weinstein Monofilaments, 2PD, and SFO, based on published normative/reference thresholds or established cut-offs (i.e., not based on comparison with the contralateral limb). All other motor and somatosensory outcomes were analysed as continuous/ordinal measures to quantify pre–post change rather than to classify impairment status. The assessment battery focused primarily on tactile and haptic domains, while proprioceptive components were addressed within the intervention but were less directly represented in the outcome measures.

### 2.4 Statistical analysis

Data analysis was conducted using SPSS (v.25, SPSS Inc., Chicago, IL, USA). Data were examined for normality using the Shapiro–Wilk test. Descriptive statistics are presented as number (n) and percentage (%), and as means and standard deviation or as median and interquartile range, depending on whether variables followed a parametric or non-parametric distribution. COPM was the primary outcome. All other measures were considered secondary outcomes and were analysed to characterise exploratory patterns of change in UL motor performance and somatosensory processing. To address aims (a) and (b), pre–post comparisons were performed using paired t tests for parametric variables and Wilcoxon signed rank tests for non-parametric quantitative variables. Depending on the distribution of the data, Cohen's d or r were used to interpret the magnitude of the differences (i.e., an effect size of less than 0.2 reflects a negligible mean difference; between 0.2 and 0.5, a small difference; between 0.5 and 0.8, a moderate mean difference; and 0.8 or greater, a large difference) (57). No formal adjustment for multiple comparisons was applied. The level of significance was set at *p* < 0.05.

## 3 Results

Given the exploratory single-arm pre–post design, percent changes are reported descriptively, and *p*-values/effect sizes are provided to contextualise the observed pre–post differences (Tables 2, 3).

### 3.1 Baseline characteristics of the participants

**Figure 2:**
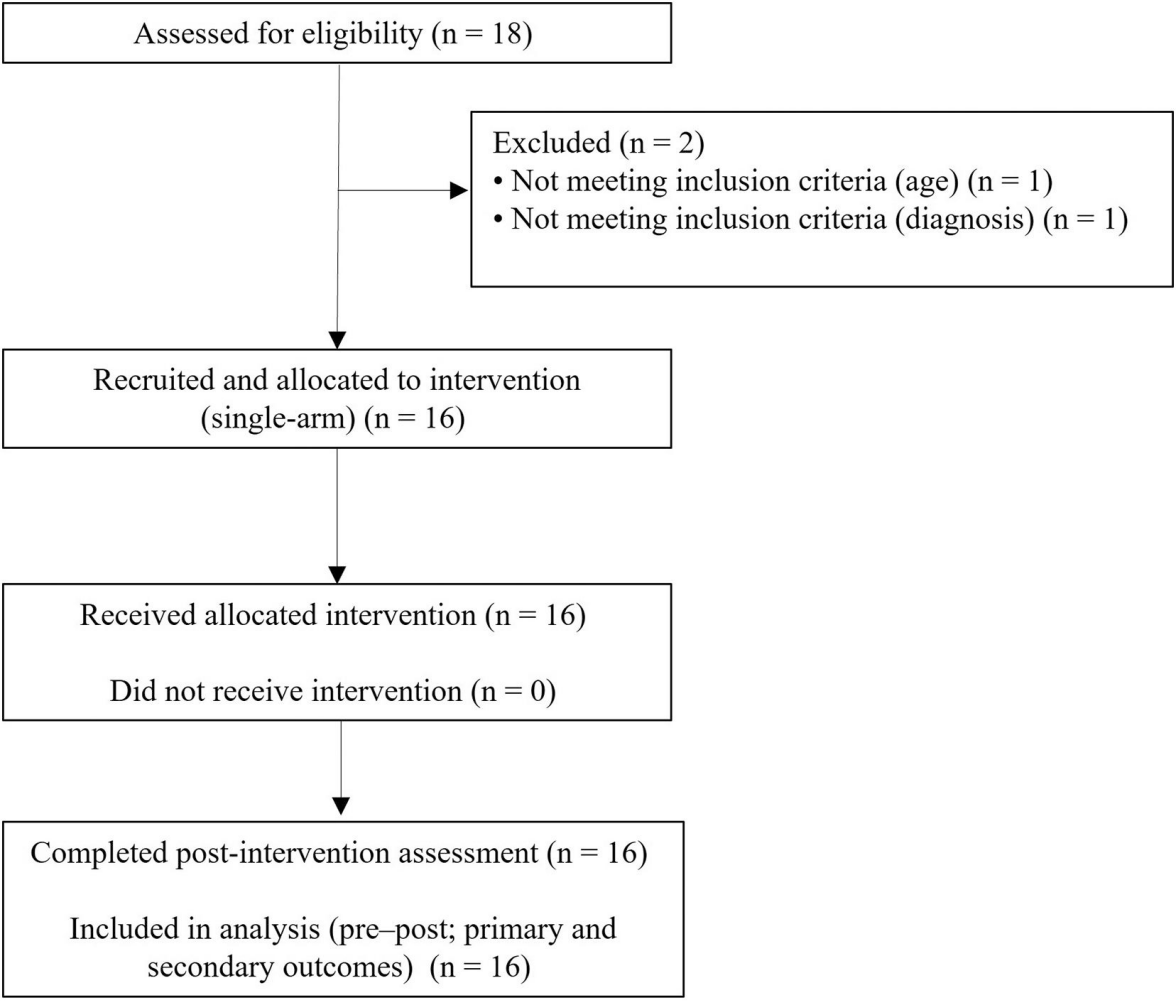
Participant flow diagram. Exploratory single-arm pre–post study of an intensive somatosensory activity-based intervention targeting the more-affected upper limb in children with unilateral cerebral palsy.

The participant flow diagram is shown in Figure 2. Eighteen children were assessed for eligibility and two were excluded for not meeting the inclusion criteria, resulting in a final sample of 16 children. All participants completed the intervention and the post-intervention assessment (retention: 100%; attrition: 0%). The sample included nine girls and seven boys (mean age 8.53 years, SD 2.33). 62.5% (*n* = 10) showed more impairments on the right side of the body and 37.5% (*n* = 6) on the left side. MACS levels were distributed as follows: level I = 6, level II = 8 and level III = 2.

**Figure 3:**
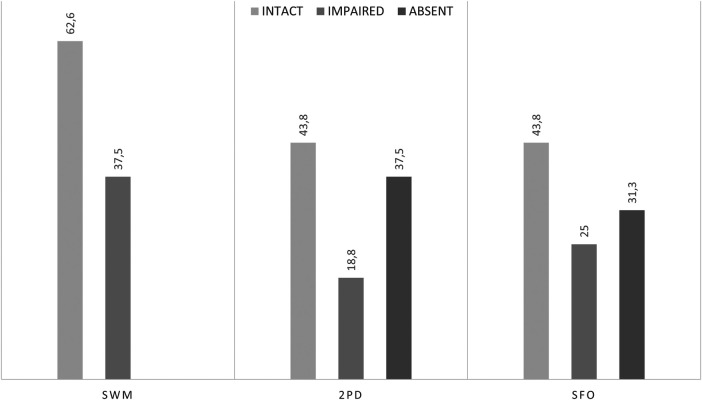
Baseline characteristics of the participants. Frequency distribution of scores on four of the somatosensory tests (%). SWM: Semmes–Weinstein monofilaments; 2PD: two points discrimination; SFO: stereognosis of familiar objects.

Regarding somatosensory processing of the more-affected hand, deficits were defined using published normative/reference thresholds or established cut-offs (not by comparison with the less-affected limb). Specifically, tactile registration deficits were identified using Semmes–Weinstein monofilaments (threshold above the normative registration level: 2.83), tactile spatial perception deficits using 2PD (intact ≤5 mm, impaired 6–10 mm, absent >10 mm), and stereognosis deficits using SFO (intact = 6 objects correctly identified, damaged = 4–5, absent ≤3). Using these criteria, 37.5% (*n* = 6) showed deficits in tactile registration, 56.3% (*n* = 9) in tactile spatial perception, and 56.3% (*n* = 9) had reduced performance in stereognosis (Figure 3). Corresponding baseline test values are reported in Table 3, and detailed criteria and references are provided in Supplementary Material 3.

### 3.2 Intensive somatosensory activity-based intervention (ISABI)

Children did a mean of 29.62 h (SD = 0.80) of practice in the clinical setting and a mean of 5.50 h (SD = 0.81) of practice at home, making a total mean of 35.12 h (SD = 1.31) of intervention. This corresponds to 98.7% adherence for clinic-based practice (29.62/30 planned hours), 91.7% adherence for home practice (5.50/6 planned hours), and 97.6% adherence overall (35.12/36 planned hours).

Regarding home practice, five children did not complete all planned sessions: three children missed the activities for one weekend (2 h each), and two children missed one day of a weekend (1 h each). The remaining 11 children completed all home-practice hours. Regarding clinic attendance, three children missed one clinic day (2 h) due to illness; in addition, one child arrived late for one session, resulting in a small partial dose reduction.

#### 3.2.1 Participation-related goals

Families reported a total of 32 problems in their children's daily occupations, two problems per family. They were distributed across the three main occupational areas, and the most frequently experienced problems were those related to the area of self-care (81.25%), followed by leisure (12.50%) and productivity (6.25%) (Table 1).

**Table 1:** Occupational performance problems by area and sub-area identified by families.

Occupational areas	Occupational sub-areas	Occupational performance problems	Frequency (*n*)	Percentage (%)
Self-care *n* = 26	Personal Care	Feeding	8	25
		Clothing	12	37.5
		Personal hygiene	6	18.75
Productivity *n* = 2	Play/School	Homework	2	6.25
Leisure *n* = 4	Quiet recreation	Playing cards	1	3.125
		Playing with stickers	1	3.125
	Active recreation	Jumping rope	2	6.25

Within the main area of self-care, all the problems identified were within the subarea of personal care. Most of the problems reported by the families were related to dressing (37.5%), especially opening and closing buttons and zippers. Putting on and taking off shirts was also mentioned. 25% reported problems related to feeding, the most frequent being the handling of cutlery. One family reported problems when opening plastic bags (such as pastries or potato chips). In total, 18.75% of the personal care problems were related to personal hygiene, the most frequent being ponytailing. Brushing teeth and toileting were also mentioned.

Within the area of productivity, all the problems identified were related to the sub-area of school performance (6.25%), namely using the pencil sharpener and stabilizing the paper when writing or painting.

Finally, in the area of leisure, the problems identified were playing cards, playing with stickers and jumping rope.

Following ISABI, the COPM indicated observable increases in both children's performance and family satisfaction. Children's performance scores increased by 71.47%, from 6.03 (SD = 2.88) pre-intervention to 10.34 (SD = 4.61) post-intervention (*p* < 0.001; d = 0.935). Family satisfaction scores increased by 62.51%, from 8.43 (SD = 4.09) to 13.70 (SD = 3.93) (*p* < 0.001; d = 1.344). Full results are presented in Table 2.

**Table 2:** Measures of motor performance and participation before and after intensive somatosensory activity-based intervention. .

	UCP (*n* = 16)			
	Pre	Post		
Participation	Mean (SD)	Mean (SD)	*p* value	ES
COPM				
Performance	6.03 (2.88)	10.34 (4.61)	<0.001^b^	0.935^d^
Satisfaction	8.43 (4.09)	13.70 (3.93)	<0.00^b^	1.344^d^
Motor Performance				
JTHFT (Seconds)	277.06 (179.43)	242.77 (178.87)	0.017^a^	0.616^c^
Box and Block Test Number of cubes)	18.75 (7.91)	21.00 (10.01)	0.028^b^	0.225^d^

UCP: unilateral cerebral palsy; JTHFT: Jebsen–Taylor Hand Function Test; COPM: Canadian Occupational Performance Measure; SD: standard deviation; ES: effect size.

a Wilcoxon test.

b Student *t* test.

c r value.

d Coheńs d.

#### 3.2.2 Motor performance

Observable improvements in dexterity, speed and coordination of the more-affected UL were noted following ISABI. On the JTHFT, performance improved by 12.38%, reflected by a reduction in total completion time from 277.06 s (SD = 179.43) pre-intervention to 242.77 s (SD = 178.87) post-intervention (*p* = 0.017; r = 0.616). Manual dexterity, assessed with the BBT, increased by 12%, from 18.75 blocks (SD = 7.91) to 21.00 blocks (SD = 10.01) (*p* = 0.028; d = 0.225). Full results are reported in Table 2.

#### 3.2.3 Somatosensory processing

Tactile registration (Semmes–Weinstein monofilaments) showed no change, with median values remaining at 2.83 pre- and post-intervention (*p* = 0.138; r = 0.383). For SPL, median performance for the 1st, 2nd and 5th fingers reached the normative range (0 mm) post-intervention, with the 2nd finger improving from 2.00 mm to 0.00 mm (*p* = 0.040; r = 0.530). 2PD showed a 33% improvement (median 6–4 mm), with the post-intervention median in the “intact” range (≤5 mm), although this did not reach statistical significance (*p* = 0.230; r = 0.310). DS increased by 9.10% (median 22–24 correct trials; *p* = 0.015; r = 0.627), and graphaesthesia increased by 33.33% (median 3–4; *p* = 0.019; d = 0.898).

With regard to haptic perception, no observable changes were found in the SFO or in Manual Form Perception Test. Texture perception increased by 33% (median 3–4 correct textures; *p* < 0.001; r = 0.901), and functional sensibility (BBT without vision) increased by 18.52% (median 13.50–16 blocks; *p* = 0.002; r = 0.807). Full results are reported in Table 3.

**Table 3:** Measures of somatosensory processing before and after intensive somatosensory activity-based intervention.

	UCP (*n* = 16)			
	Pre	Post		
Somatosensory proccessing	Median (IQR)	Median (IQR)	*p* value	ES
SWM (Monofilament)	2.83 (2.44–3.22)	2.83 (2.38–3.22)	0.138^a^	0.383^c^
Single Point Localization (Milimetres)				
1st finger	1.50 (0–10)	0 (0–4.75)	0.213^a^	0.322^c^
2nd finger	2 (0–39.50)	0 (0–0.75)	0.040^a^	0.530^c^
5th finger	0.50 (0–9.75)	0 (0–0)	0.141^a^	0.381^c^
Hipotenar	12.50 (7–16.50)	9 (2.25–15.75)	0.434^b^	0.384^d^
Two Points Discrimination (millimetres)	6 (4–12)	4 (4–10.50)	0.230^a^	0.310^c^
Double Simultaneous (Number of correct trials)	22 (16.25–24)	24 (21.25–24)	0.015^a^	0.627^c^
Graphaesthesia	3 (2–4)	4 (4–5)	0.019^b^	0.898^d^
Stereognosis of Familiar Objects				
Number of correct objects	5 (2.25–6)	4.50 (3–6)	1.00^a^	0.000^c^
Seconds of the correct objects	30.91 (17.98–58.88)	24.33 (16.44–33.74)	0.179^a^	0.347^c^
Manual Form Perception Test				
Number of correct forms	2 (0–3)	2 (1–4)	0.096^a^	0.429^c^
Seconds of the correct forms	25.31 (0–48.86)	31.88 (13.11–66.50)	0.063^a^	0.481^c^
Texture Perception (Number of correct textures)	3 (2–3)	4 (3.25–4)	< 0.001^a^	0.901^c^
BBT without visión (Number of cubes)	13.50 (8–14.75)	16 (11.75–18.00)	0.002^a^	0.807^c^

UCP: unilateral cerebral palsy; SWM: Semmes–Weinstein monofilaments; BBT: box and block test; IQR: interquartile range; ES: effect size.

a Wilcoxon test.

b Student *t* test.

c r value.

d Coheńs d.

## 4 Discussion

This study describes the implementation of ISABI targeting the more-affected UL in children with UCP. In this exploratory single-arm pre–post study, changes were observed in occupational performance goals, UL motor performance, and somatosensory processing following the intervention. These findings should be interpreted cautiously and are intended to inform hypothesis generation and the design of future adequately powered randomised trials.

Most of the occupational problems identified in the children were distributed in the main occupational area of self-care (81.25%), followed by leisure (12.50%) and finally productivity (6.25%). Following ISABI, the COPM scores indicated increases in children's daily occupational performance and in family-reported satisfaction. These observations are broadly consistent with findings reported by McLean et al (37). However, unlike the somatosensory discrimination intervention evaluated in their study, the ISABI model did not directly train the family-selected goals. We hypothesise that enhancements in somatosensory processing in the more-affected UL may have supported more effective bimanual engagement in ADLs, which could help explain the observed changes in goal-related performance. This hypothesis is consistent with literature indicating that somatosensory function contributes to hand use, motor control, and performance in daily activities across populations (5, 6, 17, 18, 58, 59). This proposed mechanism remains speculative and should be examined in future adequately powered randomised trials, some of which are now underway in children and adolescents with UCP (60). In addition, collaborative goal-setting with families can orient attention and effort towards meaningful activities, potentially reinforcing practice beyond the therapy context (61).

In terms of motor performance, group-level pre–post increases were observed on the JTHFT (12.38%) and the BBT (12%). These patterns are broadly consistent with Kuo et al. (62), who reported increases in both unimanual and bimanual motor performance following 90 h of intervention (82 h bimanual + 8 h tactile training). McLean et al. (37) similarly found better bimanual performance after a somatosensory discrimination training, although unimanual performance did not change. By contrast, Auld et al. (38) did not include motor outcome measures, precluding conclusions regarding motor performance in their study.

Regarding tactile registration, it seems that, despite the different treatment doses reported in the available studies [90 h in Kuo et al. (62), 1.5 h in Auld et al. (38), 6.71 h in Hobbs et al. (33) and 35.12 h in ISABI], existing somatosensory interventions have shown limited effects on tactile registration deficits. This may reflect the fact that changes in basic sensory registration thresholds are less sensitive to short-term intervention compared to higher-level perceptual functions, which are more likely to capture improvements in the discrimination and interpretation of sensory input (35).

To assess tactile perception, five outcome measures were administered: SPL, 2PD, DS, graphaesthesia, and texture perception. Group-level pre–post increases were observed across all five measures. Of note, SPL and DS are clinically relevant: prior work has reported that more than 30% of the variance in unimanual capacity can be explained by SPL performance, and more than 30% of the variance in bimanual performance by DS outcomes (6). This has important clinical implications, as it could be predicted that improved tactile perception could facilitate improved motor function (38). Comparisons with previous studies are constrained by heterogeneity in intervention content and dose. For example, Kuo et al. (62) delivered approximately 90 h of intervention and reported significant gains in tactile perception in both groups on the Grating Orientation Task, with no changes in other perceptual domains. By contrast, Auld et al. (38) evaluated a single 1.5-hour mirror-based tactile and motor session and reported increases in tactile spatial perception. McLean et al. (37) implemented an 18-hour *sense©* training; however, their pilot did not include tactile perception outcome measures, precluding direct comparison on those domains.

Regarding stereognosis, we did not observe pre–post changes in the SFO or Manual Form Perception Test. Notably, 43.8% (7/16) of participants achieved the maximum baseline score on SFO, indicating a potential ceiling effect. Moreover, although the procedures employed show adequate inter-rater reliability and excellent test–retest reliability (12), they may be insufficiently responsive to change over time (43). These observations are consistent with Kuo et al. (62). By contrast, McLean et al. (37) reported higher median haptic object recognition scores post-intervention, although similar increases were also observed in the control group. More recently, Steinbusch et al. (63) reported gains in stereognosis and other somatosensory domains following 80–90 h of bimanual intensive functional training without enriched sensory materials. This challenges the evidence so far that, despite high doses of intervention, tactile stimuli that are applied “unintentionally” lead to very limited or no improvement in tactile performance (31). Taken together, the mixed evidence underscores the need for further research with rigorous controls, dose–response characterisation, and outcome measures with demonstrable responsiveness.

Finally, functional sensibility showed a 14.5% pre–post increase, an aspect of somatosensation described by Krumlinde-Sundholm and Eliasson (64). When vision was occluded and object manipulation relied more heavily on somatosensation, children appeared less dependent on visual guidance for successful performance, potentially reflecting greater functional use of somatosensory feedback. This interpretation is consistent with evidence that, during bimanual task performance, the preferred (manipulative) hand is guided primarily by vision, whereas the non-preferred hand typically stabilises and holds objects using somatosensory information (65).

Several limitations should be considered when interpreting these findings. First, this was an exploratory study with a small sample size, using a single-group pre–post design without a control condition or follow-up assessment; therefore, causal inference is not possible and the findings may have been influenced by selection bias, regression to the mean and practice effects. Second, inferential statistics were included to contextualise the observed pre–post changes and to explore preliminary trends; however, the study was not powered to provide definitive evidence of intervention effects. In addition, because multiple outcomes were examined and no formal adjustment for multiple comparisons was applied, the risk of type I error is increased and *p* values—particularly for secondary outcomes—should be interpreted with caution. Third, the procedures used to assess texture perception, SPL and manual form perception have not been fully standardised for children with UCP, which may limit measurement precision and responsiveness to change. Future studies should incorporate a control condition, blinded outcome assessment and longer-term follow-up to examine retention, and may also explore how somatosensory activity-based training can be integrated with established rehabilitation approaches and whether combined programmes are acceptable and beneficial for children and families.

## 5 Conclusion

In this exploratory single-arm pre–post study, ISABI was associated with observable pre–post improvements in goal-related occupational performance (COPM children's performance and family satisfaction), UL motor performance, and selected domains of somatosensory function in children with UCP. Changes were more evident in tactile perceptual and functional sensibility outcomes than in tactile registration or stereognosis. While these findings should be interpreted cautiously given the study design, they provide preliminary signals that warrant consideration of somatosensory function in clinical assessment and intervention planning. Confirmation is required in adequately powered randomised trials with blinded assessment and longer-term follow-up.

## Data Availability

The dataset contains information from paediatric participants and cannot be shared publicly due to ethical and privacy restrictions. De-identified data required to replicate the findings reported in this manuscript are available upon reasonable request from the corresponding author (PJI) and subject to approval by the relevant institutional/ethics oversight and completion of a data use agreement.

## Supplementary material

SUPPLEMENTARY FILE 1. Somatosensory Activity-Based Intervention (ISABI) manual.

SUPPLEMENTARY FILE 2. Intensive Somatosensory Activity-Based Intervention (ISABI) theoretical principles mapped to practice.

SUPPLEMENTARY FILE 3. Motor and somatosensory outcome measures: administration, scoring, interpretation, and key references.
