# Supplementary material for: An exploratory pre–post study of an intensive somatosensory activity-based intervention on participation-related goals, motor performance and somatosensory function in children with unilateral cerebral palsy
Source: Front Pediatr. 2026 Jun 25;14:1862592. doi: 10.3389/fped.2026.1862592 (PMC13345887; doi:10.3389/fped.2026.1862592)
Supplement: SUPPLEMENTARY FILE 1. — Somatosensory Activity-Based Intervention (ISABI) manual. [file Supplementaryfile1.docx]

Supplementary Material

# Supplementary material 1: Intensive Somatosensory Activity-Based Intervention (ISABI) manual.

# This document provides a structured description of the ISABI, including delivery format, dose, session/weekly structure (stable template with theme-based variation), progression and grading (day-by-day with individualisation), weekend home practice, and a worked example of a themed week.

**A. Setting and general delivery**

Delivery format: Group-based sessions with children and interventionists seated around a table to facilitate interaction and play-based engagement. Children were divided into two age groups (6–10 years and 11–15 years). Each child worked with a trained interventionist across the programme, and the lead researcher supervised sessions to support consistency and appropriate individual task grading.

To support engagement and sustained attention, both competitive and cooperative play activities were planned, and individual play interests (obtained using Takata’s play history) were considered when selecting and adapting activities. This approach was intended to enhance motivation and help children remain engaged throughout the session.

Target limb and vision: Activities were targeted to the more-affected upper limb. Many activities were performed without vision using either (i) an eye mask, or (ii) a “magic box” that allowed children to keep their eyes open while preventing visual access to the explored stimuli.

**B. Programme dose**

Clinic dose: 2 hours per day, Monday to Friday, for three consecutive weeks (30 hours). Home dose: weekend home practice was proposed for 1 hour per day during weekends (total 6 hours).

**C. Session/weekly structure and theme-based variation**

The weekly structure (Monday–Friday) and the session domain sequence were consistent across the three intervention weeks. The exact minutes per block could vary slightly due to transitions and the needs of the group, but all planned domains were addressed each day.

***C1. Session template (domain sequence and approximate timing)***

| **Approx. time** | **Domain** | **Typical focus / notes** |
| --- | --- | --- |
| ~15 min | Tactile registration | “Wake-up” activities to prepare the arm/hand for subsequent discrimination tasks. |
| ~25 min | Tactile spatial perception | Unilateral and bilateral spatial tactile activities (primarily palm/hand). |
| ~25 min | Alternating block A | Texture perception (Mon/Wed/Fri) OR Graphaesthesia (Tue/Thu). |
| ~30 min | Alternating block B | Proprioception (Mon/Wed/Fri) OR Haptic perception / stereognosis (Tue/Thu). |
| ~25 min | Themed sensory story | Story-based activities structured around a narrated sensory story, integrating multiple somatosensory modalities (vibration, temperature, graphaesthesia, texture perception, proprioception, stereognosis), were developed over several days (see Section D). |

***C2. Week-to-week themes and materials***

Weekly themes (e.g., “Travelling into space”, “Enchanted forest”, “We become cooks”) guided the selection of materials and narratives, increasing stimulus variety, supporting motivation and sustained attention, and enabling additional task variation and, when appropriate, increased discrimination demands while retaining the same domain targets and grading logic.

**D. Progression and grading (day-by-day)**

***D1. How progression was implemented***

Progression was implemented day-by-day within each somatosensory domain by systematically manipulating predefined grading variables. Although the same activity “shell” was delivered in the group, task difficulty was individualised for each child by adjusting these variables (e.g., stimulus size, similarity, and required response precision; cue availability; number of options; and response format).

***D2. Grading variables (overview)***

Within each domain, activity difficulty was graded using domain-specific variables. Examples include: (i) stimulus parameters (size, pressure, speed, and similarity/number of choices); (ii) cue availability and calibration opportunities (e.g., visual preview, verbal labelling, intermodal or intramodal matching) with progressive withdrawal; and (iii) response demands (type and precision of response, time constraints). Concrete examples of grading variables are provided in Section F.

***D3. Tracking and individualisation***

All grading variables used in the programme were included in a domain-specific tracking sheet. For each session, the interventionist marked (e.g., with an “X”) which variables were applied for that child, supporting an “optimal challenge point” (challenging but achievable) and enabling consistent progression over time.

***D4. Illustrative composite example 1: themed sensory story (Mon–Fri)***

The themed sensory story provided a motivating narrative thread and an opportunity to integrate varied sensory inputs (e.g., temperature, vibration, diverse tactile stimuli, textures, haptic object recognition, and proprioceptive elements) within play. Across the week, access to cues and task demands were progressively adjusted as follows (illustrative composite example; not individual participant data):

| **Day** | **Story access / sensory conditions** | **Examples of task demands** |
| --- | --- | --- |
| Mon | Listen + see + sensory exploration; both hands allowed (less-affected hand first, then more-affected hand) | Introduce story elements; explore materials using visual and somatosensory input; name/recognise key items. |
| Tue | Listen + sensory exploration; vision removed; both hands allowed (less-affected hand first, then more-affected hand) | Explore and recognise story items without vision; matching/selection tasks with increased reliance on somatosensory processing. |
| Wed | Listen + sensory exploration; vision removed; more-affected hand only | Unimanual exploration with the more-affected hand; discriminate and select story items; reduced compensatory input from the less-affected hand. |
| Thu | Listen + sensory exploration; vision removed; more-affected hand only | Higher discrimination demands (e.g., increased similarity/choices); minimal cues; sustained attention to somatosensory features. |
| Fri | No audio (“story scramble”); free exploration of all story materials using somatosensory processing | Recognition/recall challenge: identify story elements through exploration; generalisation across the week’s material set. |

***D5. Illustrative composite example 2: haptic perception (stereognosis) – “Supermarket shopping”***

This example illustrates how a single stereognosis activity can be graded across days by manipulating stimulus characteristics, calibration/cue conditions, number of options, similarity between objects, and response demands. It represents one of several possible progression pathways (illustrative composite example; not individual participant data).

| **Day** | **Calibration / cue conditions** | **Main task demands (examples of graded variables)** |
| --- | --- | --- |
| Mon | Intermodal calibration (visual feedback) and intramodal calibration (sensory exploration with less-affected hand first, then more-affected hand) | Start with two very different fruits (size/shape/weight/texture). Child sees and explores with both hands (less-affected hand first, then more-affected hand). Then vision is removed and the child matches what is felt in one hand (less-affected) to the corresponding item using the other hand (more-affected). Gradually increase the number of fruits while maintaining initial visual preview. |
| Tue | Intramodal calibration only (no visual preview) | Increase number of fruits and match pairs without prior visual preview. Maintain matching format but increase similarity within pairs and number of choices. |
| Wed | No inter- or intramodal calibration | Present single fruits one at a time for identification with the more-affected hand only. Progress from highly distinct fruits to increasingly similar fruits. |
| Thu | List-guided search (more-affected hand) | Child has a “shopping list” of target fruits. Many fruits are in a box; some are targets and others are distractors. Child identifies which items belong to the list using the more-affected hand. |
| Fri | List-guided search + ordering by size (more-affected hand) | Expanded “supermarket” with many items, including multiple exemplars of each fruit in different sizes. Child finds targets and then orders small vs large exemplars for each fruit type, increasing response precision and cognitive demand. |

**E. Weekend home practice**

During weekends, families were provided with somatosensory activities and necessary materials, together with written support, and were invited to practice for 1 hour per day. To record compliance, a checklist was signed by the family and child after each weekend and returned to the therapist, indicating which activities were completed and for how long.

**F. Worked example: treatment session within the themed week “Travelling into space”**

The table below provides an example of activities used within one themed week, the grading variables used to increase or decrease task complexity and example materials. For readability, grading variables are grouped into three categories: (1) stimulus parameters, (2) calibration/cues (stimulus presentation and cue availability), and (3) response demands. Note that “calibration/cues” refers to the availability of sensory cues during stimulus presentation (e.g., visual preview, verbal labelling, intramodal calibration), and not to performance feedback provided after a response.

| **Somatosensory domain** | **Activity (name and brief description)** | **Grading variables (examples)** | **Example materials** |
| --- | --- | --- | --- |
| Tactile registration | *“Preparing for the adventure”:* using a brush, shaving foam is spread over the arm/hand as a playful “protective layer” before the space mission. | **Stimulus parameters:** Type of stimulation (brush/sponge/spiky ball/foam/paint); who provides stimulation (therapist vs child).  **Calibration / cues (stimulus presentation):** Visual attention to stimulated area (eyes open) vs eyes closed; verbal labelling of stimulated areas vs no labelling.  **Response demands:** The child identifies the stimulated area (naming/pointing), progressing from general to more precise location and, when required, to discrimination tasks involving identification of the exact stimulated area. | Brushes, sponges, spiky balls, body cream, shaving foam, finger paint, exfoliating scrub. |
| Tactile spatial perception | *“Catching meteorites falling from space”:* stickers (“gomets”) of different sizes are placed on the arm/hand and the child must locate them. | **Stimulus parameters:** Stimulus size/thickness; pressure exerted when applying the stimulus.  **Calibration / cues (stimulus presentation):** Tactile feedback from the less-affected hand is permitted to assist in locating the stimulus vs restrict; use of a drawn hand template vs no template.  **Response demands:** (a) discriminate one vs two points; (b) indicate general area vs exact location; (c) point directly on the hand vs point on a drawn template. | Stickers of different sizes/thickness; brushes of different thickness; paint; stampers. |
| Graphaesthesia | *“Guessing the aliens’ messages”:* symbols are traced on the palm and the child identifies them to decode a message. | **Stimulus parameters:** Stimulus size; pressure; application speed; symbol complexity.  **Calibration / cues (stimulus presentation):** Show the symbol options visually vs no preview; verbal labelling of options vs none.  **Response demands:** Number of options; response format: point to correct option; reproduce the symbol on the more-affected hand with the less-affected hand; reproduce on a drawn hand template. | Symbol cards (lines, shapes, letters, numbers); pencil with eraser/tipped tool for tracing. |
| Texture perception | *“Finding our astronaut suit”:* children choose a suit by exploring fabrics with different characteristics. | **Stimulus parameters:** Number and similarity of textures (start with very different textures; add more similar textures; increase choices).  **Calibration / cues (stimulus presentation):** intermodal (visual + verbal description of texture features) vs intramodal (sensory exploration with less-affected hand first, then more-affected hand); progressively withdraw cues.  **Response demands:** match an object held in the less-affected hand with the same object using the more-affected hand vs identify with the more-affected hand only; optionally describe texture characteristics verbally. | Textured cylinders (feathers, Velcro, wool, sandpaper, leather, artificial grass); textured boards; fabrics (silk, corduroy, cotton); sensory dominoes. |
| Haptic perception (stereognosis) | *“Preparing the suitcase for the moon”:* each child searches for and identifies objects needed for the trip. | **Stimulus parameters:** Number and similarity of objects (start with few and very different; gradually increase and include more similar items); object type (meaningful everyday objects, 2D/3D shapes).  **Calibration / cues (stimulus presentation):** inter-modal (visual/verbal) and/or intramodal (sensory exploration with less-affected hand first, then more-affected hand), progressively withdrawn.  **Response demands:** match an object held in the less-affected hand with the same object using the more-affected hand; identify a single object with the more-affected hand; search for an object fully covered by distractor material (e.g., in a bag of chickpeas) with/without identification. | Everyday objects (cutlery, combs, keys, pens, jars, fruits); 2D/3D pieces; bags with fillers (chickpeas, beans, pasta, straw, paper) to hide objects. |
| Proprioception | *“Finding the moon”:* proprioceptive discrimination tasks focusing on joint position and object weight perception without visual input. | **Stimulus parameters:** Position differences (joint angles/postures); object weight differences; increase in the number of options and similarity between options (from clearly distinct to increasingly similar).  **Calibration / cues (stimulus presentation):** Visual preview vs no visual input; verbal cues vs no verbal cues; use of the less-affected hand for comparison (bimanual matching) vs unimanual; progressive withdrawal of cues.  **Response demands:** (a) joint position matching (reproduce a demonstrated wrist/hand posture in the less-affected hand with the more-affected hand without vision);  (b) force grading (apply “soft/medium/strong” force to reach a target); and/or  (c) discrimination and ordering tasks: (i) identify objects of different weights with similar size (heavier vs lighter); (ii) identify objects of similar weight with different size/shape; (iii) order objects according to weight or size. | Objects of identical size with different weights; balls/spheres of different weights; sponges/foam of different densities; optional force targets (e.g., marked squeeze objects). |

Note: When selecting object size and type, consider the child’s in-hand manipulation skills and adjust materials accordingly.
