# Supplementary material for: An exploratory pre–post study of an intensive somatosensory activity-based intervention on participation-related goals, motor performance and somatosensory function in children with unilateral cerebral palsy
Source: Front Pediatr. 2026 Jun 25;14:1862592. doi: 10.3389/fped.2026.1862592 (PMC13345887; doi:10.3389/fped.2026.1862592)
Supplement: SUPPLEMENTARY FILE 2. — Intensive Somatosensory Activity-Based Intervention (ISABI) theoretical principles mapped to practice. [file Supplementaryfile2.docx]

Supplementary Material

# Supplementary material 2: Intensive Somatosensory Activity-Based Intervention (ISABI) theoretical principles mapped to practice.

# This table summarises the main theoretical principles underpinning the ISABI programme and how they were translated into practice, with concrete examples.

| **Principle / framework** | **Brief definition** | **How translated into practice (operationalisation)** | **Concrete activity examples (ISABI)** |
| --- | --- | --- | --- |
| *Specially designed somatosensory activity-based approach* | The intervention is purpose-built to train multiple somatosensory domains, clinically organised into three related levels: tactile processing, proprioceptive sensitivity, and higher-order cortical/haptic functions. | Each session targets multiple somatosensory domains following a consistent sequence (registration → unilateral/bilateral spatial perception → alternating graphaesthesia and texture perception → alternating haptic perception and proprioception → themed sensory story). | The session structure and representative activities by domain are detailed in Supplementary Material 1. |
| *Principles of motor learning and neuroplasticity* | Repetition, high intensity of practice, graded challenge, feedback, and reward are used to facilitate learning-dependent plasticity through structured practice with increasing complexity. | High-dose schedule (30 h clinic over 3 weeks plus 6 h home practice). Activities are graded using predefined complexity variables, with corrective feedback provided to guide error adjustment and weekly rewards (mission-based “prizes”) used to reinforce engagement. | Examples of grading variables and representative activities are illustrated in Supplementary Material 1. |
| *Transfer-enhanced sensory training principles (SENSe©-type elements)* | Practice is designed to enhance transfer by using varied stimuli, attentive exploration without vision, anticipation trials and calibration (intra- and cross-modal) with performance feedback. | Somatosensory activities incorporated attentive exploration without vision (using a mask or “magic box”), preceded by practice trials, with calibration supported by visual input and the dominant hand, consistent performance feedback to support error correction. | Representative examples illustrate how key elements of sense-based training are incorporated into somatosensory activities across domains in practice, including a sensory story and a stereognosis task (“Supermarket shopping”), as detailed in Supplementary Material 1. |
| *Visually Enhanced Touch (VET effect)* | Early visual attention to the hand is used to facilitate tactile processing before transitioning to no-vision conditions. | For new activities, an initial phase involved visual attention to the stimulated area, the explored object, or hand position; visual feedback was then progressively reduced and removed as discrimination demands increased. | Across activities, visual input (eyes open attending to the stimulated area vs. eyes closed) and response demands (e.g., naming or identification) were systematically graded (Supplementary Material 1). |
| *Occupational Therapy Practice Framework* | Play-based, meaningful activities within a supportive context are used to sustain motivation and attention and to support participation-oriented practice. | Group table-based sessions; themed weeks (e.g., “Travelling into space”); cooperative and competitive games; activities adapted to children’s play interests (Takata play history). | Themed-week structure with domain-specific activity examples aligned within a common storyline is illustrated through the “Travelling into space” worked example in Supplementary Material 1. |
| *Empowerment through the use of language* | Consistent positive language is used to reduce negative labelling and promote engagement with the more-affected upper limb. | Therapists replace negative expressions (e.g., “sleeping/bad hand”) with positive terminology (e.g., “powerful hand”) during instruction and feedback. | Therapist communication throughout games/tasks consistently refers to the more-affected side in positive terms. |
| *Collaborative functional goal setting with families* | Family-identified goals guide meaningful, participation-oriented outcomes and increase relevance for the child’s daily life. | Two functional goals are identified with each family at baseline using the Canadian Occupational Performance Measure (COPM); outcomes reflect pre–post changes in performance and satisfaction for these goals. | Participation-related goals identified using the COPM (e.g., dressing, feeding, hygiene) are rated pre–post for performance and satisfaction. |
| *Emotion-facilitated learning* | Emotional engagement supports attention, motivation and learning. In ISABI, this is fostered through the therapist’s stance and communication style as well as the session narrative. | Interventionists deliberately use an empathic, energetic and motivating communication style (voice, facial expression, gesture, pacing/flow) to sustain children’s engagement, encourage persistence, and keep attention directed to somatosensory challenges. Themed narratives and creative delivery are used to maintain a consistent “storyline” and positive affect across sessions. | Themed narrative elements embedded within activities (e.g., sensory story contexts and role-based tasks) are used to support engagement and emotionally facilitated learning, as illustrated in Supplementary Material 1. |
