# Supplementary material for: An exploratory pre–post study of an intensive somatosensory activity-based intervention on participation-related goals, motor performance and somatosensory function in children with unilateral cerebral palsy
Source: Front Pediatr. 2026 Jun 25;14:1862592. doi: 10.3389/fped.2026.1862592 (PMC13345887; doi:10.3389/fped.2026.1862592)
Supplement: SUPPLEMENTARY FILE 3. — Motor and somatosensory outcome measures: administration, scoring, interpretation, and key references. [file Supplementaryfile3.docx]

Supplementary Material

# Supplementary material 3: Motor and somatosensory outcome measures: administration, scoring, interpretation, and key references.

This table provides a concise description of administration and scoring procedures for the motor and somatosensory outcome measures used in the current study. Detailed procedures for the somatosensory assessment battery are available in a previously published protocol (main manuscript reference 3), and all citations follow the Vancouver numbering of the main manuscript.

| **Outcome measure** | **Construct / domain** | **Limb(s) assessed** | **Administration (brief)** | **Scoring / scale (direction)** | **Key psychometric notes (brief)** | **Impairment classification used in this study** | **Key references** |
| --- | --- | --- | --- | --- | --- | --- | --- |
| Box and Block Test (BBT) | Unilateral gross manual dexterity | Less-affected hand first, then more-affected hand | Child transfers blocks from one compartment to the other for 60 s; total blocks transferred recorded. | Number of blocks in 60 s. Higher = better dexterity. | Reported good reliability for children with cerebral palsy. | Not used to classify impairment; analysed as a continuous outcome (pre–post change). | (53,56) |
| Jebsen–Taylor Hand Function Test (JTHFT; modified) | Timed unimanual hand function relevant to ADL | Less-affected hand first, then more-affected hand | Modified protocol: writing item omitted; each item max 120 s; total time across 6 items recorded. | Total time (s) for 6 items. Lower = better performance. | Reported good reliability for children with cerebral palsy; modified protocol previously published. | Not used to classify impairment; analysed as a continuous outcome (pre–post change). | (54, 55, 56) |
| Somatosensory assessment battery (general procedures) | Multi-domain somatosensory funtion | Less-affected hand first, then more-affected hand (exceptions noted below) | Tests conducted on the palm; performed without vision. Practice trials on the less-affected hand before scoring (2 trials: eyes open then eyes closed), except Stereognosis of Familiar Objects and Texture Perception (no practice trials). | See individual tests below. | Battery selected following evidence-based recommendations; detailed procedures previously described. | Reference-based impairment classification was applied only for SWM, 2PD and SFO (see specific rows). | (3, 12, 43) |
| Semmes–Weinstein Monofilaments (SWM) | Tactile registration / detection threshold | Less-affected hand first, then more-affected hand | Complete 20-filament kit. Tested distal pads of thumb, index, fifth digit, and hypothenar eminence. Start with normative registration threshold filament (2.83); apply 3 times per site in pseudo-random order; affirmative if ≥1/3 correct. Record lowest filament detected across tested sites (incl. sham trial within a set). | Filament threshold (log units). Lower = better registration. | Clinimetric recommendations and reproducibility for tactile assessments discussed in the literature; protocol described previously. | Yes. Impairment defined using published normative/reference registration thresholds and protocol criteria (not contralateral comparison). | (3, 43) |
| Single Point Localization (SPL) | Tactile spatial localisation (unilateral) | Less-affected hand first, then more-affected hand | Standard-pressure marker pen stimulates the same sites as SWM in pseudo-random order. Child indicates the stimulated location with the opposite hand; distance between stimulus and indicated point measured. | Distance error (mm). Lower = better localisation. | Included within evidence-based somatosensory assessment recommendations; protocol described previously. | No. Analysed as pre–post change; no reference-based impairment classification applied in this study. | (3) |
| Two-Point Discrimination (2PD) | Tactile spatial discrimination (unilateral) | Less-affected hand first, then more-affected hand | Two-point discriminator applied to index fingertip pulp with pressure to skin blanching. Start 4 mm and decrease/increase (2–10 mm). Record minimal distance with 5 consecutive correct responses. | Minimal distance (mm). Lower = better discrimination. | High reliability for tactile assessments reported; protocol described previously. | Yes. Classification followed published cut-offs: intact ≤5 mm; impaired 6–10 mm; absent >10 mm. | (3) |
| Double Simultaneous stimulation (DS) | Bilateral tactile processing / extinction | Bilateral (both hands within each trial) | Suprathreshold tactile stimuli delivered individually and simultaneously across sites on both hands in pseudo-random order. Child reports one vs two hands and identifies stimulated area(s). | Correct responses out of 24. Higher = better bilateral processing. | Recommended within comprehensive somatosensory assessment approaches; protocol described previously. | No. Analysed as pre–post change; no reference-based impairment classification applied in this study. | (3, 43) |
| Graphaesthesia | Spatiotemporal tactile perception | Less-affected hand first, then more-affected hand | Administered following the standardised procedure described in the referenced protocol; raw scores used for analysis. | Raw score sum (max 6 in protocol): 2 = correct, 1 = partially correct, 0 = incorrect per design. Higher = better. | Included within recommended somatosensory domains; protocol described previously. | No. Analysed as pre–post change; no reference-based impairment classification applied in this study. | (3) |
| Stereognosis of Familiar Objects (SFO) | Haptic object recognition | More-affected hand only | Examiner selects 6 objects from a set of 12 (including paired and non-paired objects). Child identifies objects by touch; number correct and time recorded. | Number correct (0–6) and time (s). Higher correct and lower time = better. | Protocol described previously; tactile assessment clinimetrics discussed in the literature. | Yes. Classification followed published cut-offs: intact = 6 correct; impaired/damaged = 4–5 correct; absent ≤3 correct. | (3, 12) |
| Manual Form Perception Test (MFPT) | Haptic form perception / matching | Less-affected hand first, then more-affected hand | Child palpates 5 shapes with one hand and identifies matching shapes with the other. Number correct and time recorded (raw scoring). | Number correct (0–5) and time (s). Higher correct and lower time = better. | Included within recommended haptic domains; protocol described previously. | No. Analysed as pre–post change; no reference-based impairment classification applied in this study. | (3) |
| Texture Perception | Modality-specific tactile discrimination (texture) | More-affected hand only | Child recognises 4 textures presented one at a time (5–10 s exploration each), then selects the matching texture from a panel. Number correct and time recorded. | Number correct (0–4) and time (s). Higher correct and lower time = better. | Pragmatic clinical measure described in the protocol, given the limited availability of standardised options in UCP | No. Analysed as pre–post change; no reference-based impairment classification applied in this study. | (3) |
| Functional sensibility (BBT without vision) | Dexterity without visual feedback (functional tactile use) | Less-affected hand first, then more-affected hand | BBT performed with vision occluded; child transfers blocks for 60 s. | Number of blocks in 60 s. Higher = better functional sensibility. | Conceptualised as dexterity without vision as described in the literature; operationalised via BBT without vision. | No. Analysed as a continuous outcome (pre–post change); no reference-based impairment classification applied in this study. | (3, 65) |

**
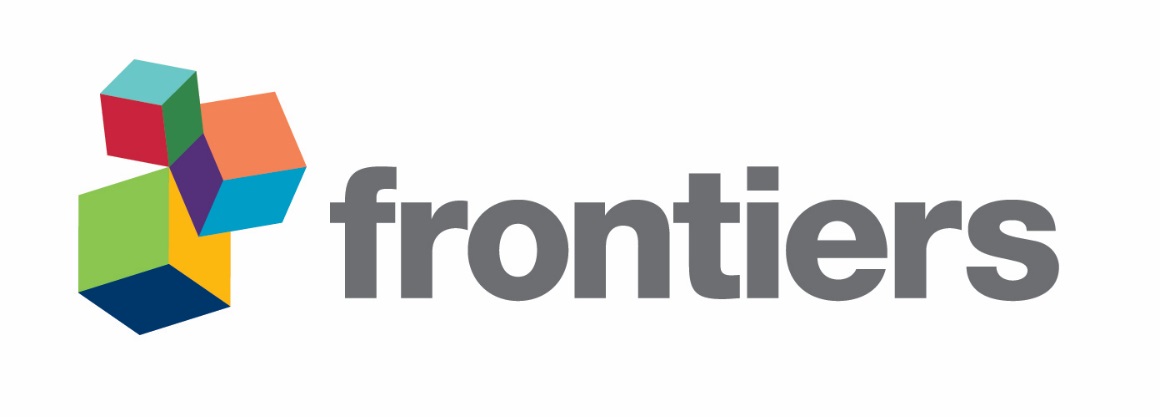
**
